# Noninvasive Estimation of Tumor Interstitial Fluid Pressure from Subharmonic Scattering of Ultrasound Contrast Microbubbles

**DOI:** 10.3390/bios13050528

**Published:** 2023-05-08

**Authors:** Yun Wang, Huimin Lu, Laixin Huang, Deyu Li, Weibao Qiu, Lingling Li, Gang Xu, Min Su, Jianhua Zhou, Fei Li

**Affiliations:** 1 Department of Ultrasound, Sun Yat-sen University Cancer Center, State Key Laboratory of Oncology in South China, Collaborative Innovation Center for Cancer Medicine, Guangzhou 510060, China; 2 Paul C. Lauterbur Research Center for Biomedical Imaging, Shenzhen Institutes of Advanced Technology, Chinese Academy of Sciences, Shenzhen 518055, China; 3 Department of Critical Care Medicine, Nanjing Drum Tower Hospital, Affiliated Hospital of Nanjing University Medical School, Nanjing 210008, China; 4 Key Laboratory for Biomechanics and Mechanobiology of the Ministry of Education, Beijing Advanced Innovation Centre for Biomedical Engineering, School of Biological Science and Medical Engineering, Beihang University, Beijing 100191, China; 5 Liver Transplant Center, Organ Transplant Center, West China Hospital of Sichuan University, Chengdu 610041, China; 6 Laboratory of Liver Transplantation, Key Laboratory of Transplant Engineering and Immunology, West China Hospital of Sichuan University, Chengdu 610093, China

**Keywords:** interstitial fluid pressure, subharmonic scattering, ultrasound contrast agent microbubbles

## Abstract

The noninvasive estimation of interstitial fluid pressure (IFP) using ultrasound contrast agent (UCA) microbubbles as pressure sensors will provide tumor treatments and efficacy assessments with a promising tool. This study aimed to verify the efficacy of the optimal acoustic pressure in vitro in the prediction of tumor IFPs based on UCA microbubbles’ subharmonic scattering. A customized ultrasound scanner was used to generate subharmonic signals from microbubbles’ nonlinear oscillations, and the optimal acoustic pressure was determined in vitro when the subharmonic amplitude reached the most sensitive to hydrostatic pressure changes. This optimal acoustic pressure was then applied to predict IFPs in tumor-bearing mouse models, which were further compared with the reference IFPs measured using a standard tissue fluid pressure monitor. An inverse linear relationship and good correlation (r = −0.853, *p* < 0.001) existed between the subharmonic amplitude and tumor IFPs at the optimal acoustic pressure of 555 kPa, and pressure sensitivity was 1.019 dB/mmHg. No statistical differences were found between the pressures measured by the standard device and those estimated via the subharmonic amplitude, as confirmed by cross-validation (mean absolute errors from 2.00 to 3.09 mmHg, *p* > 0.05). Our findings demonstrated that in vitro optimized acoustic parameters for UCA microbubbles’ subharmonic scattering can be applied for the noninvasive estimation of tumor IFPs.

## 1. Introduction

The interstitial fluid pressure (IFP) is an important characteristic of the solid tumor microenvironment. Several studies reported a high IFP in various types of tumors, including cervical cancer, breast carcinoma, and malignant melanoma [1]. The major driving force of tumor IFPs is the microvascular pressure (MVP), which is affected by the balance of fluid exchange between the microcirculation and interstitium [2]. In tumor tissues, blood vessel irregularity and leakiness, limited lymphatic drainage, interstitial fibrosis, and a contraction of the interstitial space mediated by stromal fibroblasts contribute to an increased IFP. Moreover, tumors with a high IFP tend to be correlated with high distant metastasis and recurrence rates [3,4]. Thus, monitoring IFP during the treatment process would be beneficial to identify therapy resistance and explore individual treatment schemes [5].

The IFP can be measured by the wick-in-needle technique or its modified methods, all of which involve inserting a needle into the tumor [6]. These invasive methods that may result in complications, including hemorrhage and needle tract implantation metastasis, are not suitable for widespread application. In the past decades, many noninvasive approaches have been explored to achieve a real-time assessment of IFP. Although dynamic contrast enhanced (DCE) MRI has been proven to evaluate the IFP level and heterogeneity distribution in a tumor by evaluating the infusion of a contrast agent in the tumor tissue [7], the inconvenience, time-consuming nature, and high cost limit its real-time application. 

Ultrasound imaging, as the most convenient and portable examination, has been widely used for disease screening, diagnosis, and as a treatment guide. Contrast-enhanced ultrasonography (CEUS) utilizes blood pool ultrasound contrast agent (UCA) microbubbles to enhance the intravascular backscatter and harmonic imaging technique to achieve a high contrast-to-tissue ratio (CTR) and visualization of vascular structures [8]. The clinical UCAs such as SonoVue, Sonazoid, and Definity usually consist of compressible phospholipid-shelled bubbles filled with a high molecular weight but low diffusion gas. When subjected to an incident acoustic wave, the compressibility of the microbubble makes it subject to nonlinear oscillation and generates various harmonics (f_0_, 2f_0_, 3f_0_, etc.), subharmonics (1/2f_0_) with the frequency at half the transmission frequency (f_0_), and ultraharmonics (3/2f_0_) [9,10]. Since tissues can also generate the second, third harmonic signals, etc., tissue signals by extracting higher harmonics cannot be completely suppressed [11]. While in the tissue it is usually difficult to generate a subharmonic scattering, subharmonic imaging and three-dimensional subharmonic imaging with a higher CTR have been developed and applied to multiple organs and tumor imaging [12,13,14,15]. 

Besides the ultrasound contrast subharmonic imaging, an excellent linear relationship was observed between the microbubble’s subharmonic scattering amplitude and the ambient pressure in the surrounding fluid medium: when the ambient pressure increased from 0 to 186 mmHg, the amplitude of the subharmonic signal was decreased by 10–20 dB in vitro for several UCAs including Levovist, Optison, Definity, Sonazoid, and SonoVue [16,17,18,19,20,21]. Shi et al. demonstrated that the subharmonic amplitude grows with the increase in acoustic pressure and the generation of the subharmonic can be divided into three stages: occurrence, growth, and saturation. During the growth stage, the subharmonic component is sensitive to pressure changes [16]. For SonoVue, Xu et al. found the existence of the second growth stage with a higher pressure sensitivity than that of the first growth stage before the saturation stage [22]. The above results suggest that the subharmonic is an ideal indicator of pressure variation. Compared to the conventional B-mode image, pressure-dependent subharmonic imaging has the advantages of both enhancing CTR to improve image quality and providing information on ambient pressure distribution. Consequently, the subharmonic aided pressure estimation (SHAPE) technique to determine the pressure changes was proposed [16]. This technique has been extensively studied in in vitro experiments and demonstrated by a variety of in vivo models involving intracranial pressure, portal pressure, and intracardiac pressure [17,21,23,24,25,26]. All the subharmonic signals collected from microbubbles in the large vessels or the heart cavity suggested an inverse relationship between the subharmonic amplitude and blood pressure, and had a good performance in clinical pressure stratification [27,28,29,30]. 

Tumor IFPs can be evaluated by detecting the subharmonic signals of microbubbles in tumor microvessels at a higher driving frequency compared with those used in large vessels (2–4 MHz). Previously, Definity has been used to verify the efficacy of SHAPE at the transmitting frequency of 6.7 MHz, 10 MHz, and 8 MHz in in vitro and in vivo melanoma or breast cancer animal models [31,32]. However, Definity is not approved in Asia. Both SonoVue and Sonazoid microbubbles are approved for clinical use in China, and Sonazoid was selected in the current study because of the imaging resolution benefit from its resonance frequency (~4 MHz), which was higher than that of SonoVue (~2 MHz). Although noninvasive portal vein pressure and hepatic venous pressure gradient monitoring based on subharmonics using Sonazoid have reached their ideal progress in animal experiments and clinical studies [25,27,28], the IFP estimation of superficial tumors based on Sonazoid has not been reported yet. In this investigation, we will first use Sonazoid to explore the value and stability of high-frequency excited subharmonics for IFP evaluation in tumor models.

## 2. Materials and Methods

### 2.1. Acoustic Attenuation Measurement

**Figure 1:**
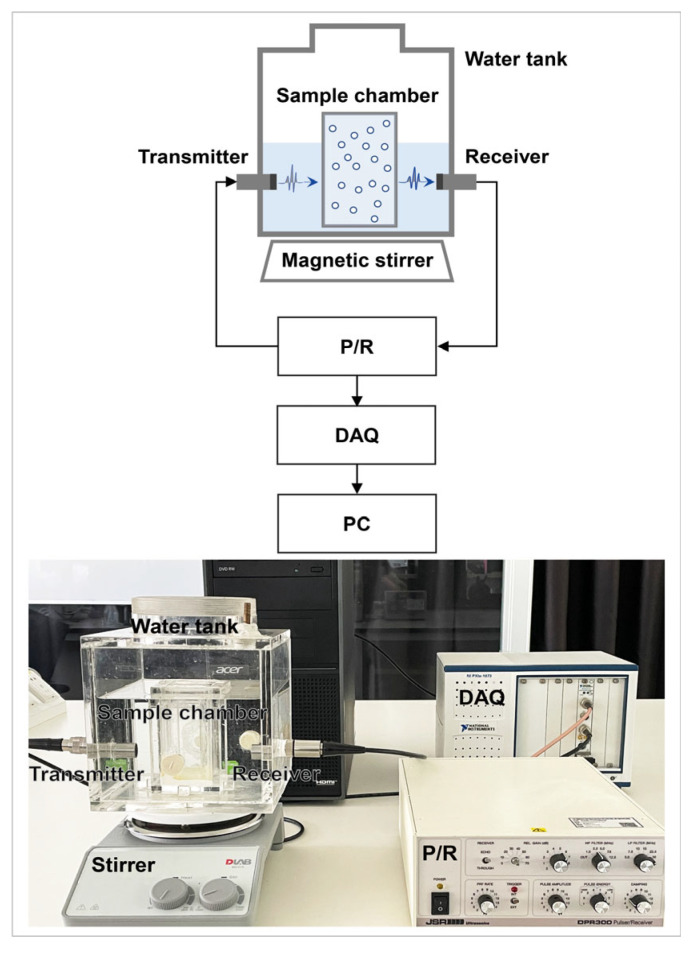
In vitro acoustic attenuation experiment setup.

The experimental setup of the acoustic attenuation measurement is shown in Figure 1 to obtain the resonance frequency of UCA Sonazoid microbubbles. Two single element flat transducers (V382-SU, Olympus, Waltham, MA, USA) were placed opposite to one another as the transmitter and receiver of the acoustic wave, respectively. The diameter of the transducer was 1.3 cm, and the center frequency was 3.5 MHz with a −6 dB bandwidth of 82.35%. The transducers were driven by an ultrasonic pulser-receiver (DPR300, JSR Ultrasinics, Pittsford, NY, USA). The microbubbles’ sample chamber was located at the center of the two transducers. The side walls of the sample chamber were made of 6 μm mylar which could allow the passing acoustic wave with minimal attenuation. After adding 200 mL of saline to the sample chamber for reference signal acquisition, 50 μL of Sonazoid (GE Healthcare, Oslo, Norway) suspension was injected into the sample chamber for sample signal acquisition. A magnetic stirrer with a low rotation speed was used to keep the microbubble suspension uniform.

The acoustic attenuation coefficient α(ƒ) can be calculated by the following formula. The acoustic path length containing UCA microbubbles in the sample chamber was z = 5 cm. The power spectra S_ref_ (ƒ) and S_sample_ (ƒ) for the reference and sample were obtained using the fast Fourier transform (FFT) of the average of 50 received signals. Furthermore, the resonance frequency of the UCA microbubbles was defined as the frequency of the maximum attenuation in the spectrum.

$$\alpha \left(ƒ\right)=\frac{1}{z}8.686\left(\ln\left(S_{\mathrm{sample}}\right)-\ln(S_{\mathrm{ref}}\right))$$

### 2.2. Subharmonic Scattering Acquisition under Different Acoustic Pressure Conditions

**Figure 2:**
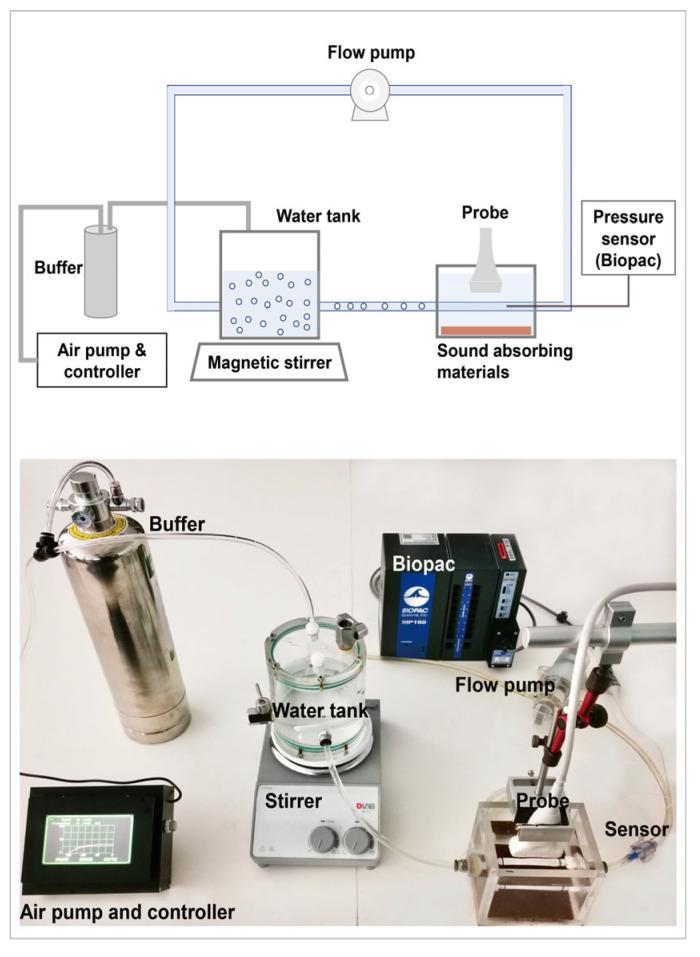
In vitro flow circulation system for acoustic scattering measurement.

The experiment setup for the acoustic scattering measurement of UCA microbubbles included a flow circulation system described in our previous study [22] and a customized ultrasound system (iNSIGHT-37CT, Saset (Chengdu) Inc., Chengdu, China) to obtain raw radio frequency (RF) data. The flow circulation system (Figure 2) contained three parts: the microbubbles’ water tank and flow pump, vessel phantom, and self-developed air pump for auto pressure regulation with a resolution of 0.1 mmHg and pressure monitor BIOPAC (MP160, BIOPAC Systems, Inc., Goleta, CA, USA). The linear array transducer with 128 elements had a center frequency of 8 MHz and a −6 dB bandwidth of 4 MHz to 12 MHz. According to previous theoretical and experimental studies, the optimal transmit frequency of the subharmonic scattering was twice the resonance frequency of the UCA microbubbles [33,34]. The transmitted frequency of the experiment was selected according to the Sonazoid’s resonance frequency from the acoustic attenuation measurement. The transmitted tone burst had 16 cycles because the microbubble needs a long pulse duration to generate stable subharmonic scattering. The acoustic pressures of the transmitted wave from the linear array were calibrated at the beam focus of 2.0 cm through a 0.5 mm needle hydrophone (Precision Acoustics, Dorset, UK) combined with a three-dimensional acoustic field measurement system (BRC8090, Shenzhen Boray Technology Co. Ltd., Shenzhen, China).

During the experiment, the transducer was positioned above the vascular phantom to ensure the focal point (focus depth 2.0 cm) in the vessel lumen based on the guide of ultrasound imaging. A piece of sound-absorbing material was located on the bottom of the water box to restrain the echo from the bottom. A bolus of 0.1 mL Sonazoid solution was injected into the water tank that contained 400 mL of 0.9% NaCl solution (4 × 10^5^ microbubbles/mL) and circulated through the system. The concentration used in the in vitro experiment was 2 × 10^−3^ μL/mL. The mean and median sizes of microbubbles were 1.46 μm and 1.29 μm. Furthermore, 95% of the microbubbles were smaller than 5 μm. Given that the IFP of a tumor is usually less than 40 mmHg, we set the ambient pressure in the range of 10–40 mmHg with a step of 10 mmHg. Beamformed RF data of 200 frames were collected and repeated three times in each acoustic pressure and ambient pressure. The microbubble solution was replenished in the dynamic flow system after each data acquisition. 

### 2.3. Tumor Models 

**Figure 3:**
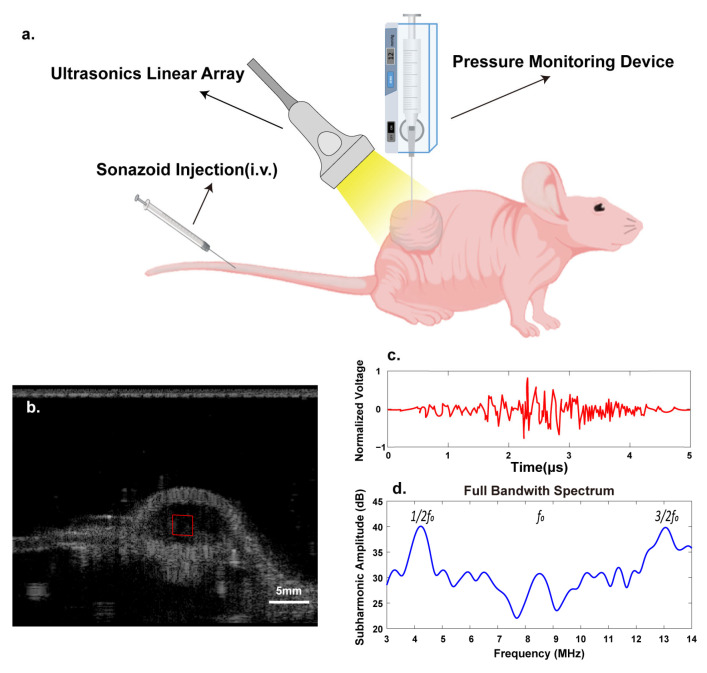
The schematic of in vivo experiment. (**a**) Ultrasonic RF data acquisition and IFP measurement. (**b**–**d**) ROI selected from reconstructed B-mode image of tumors and subharmonic wave extraction (red box, ROI selected).

This study was approved by the Institutional Animal Care and Use Committee. Female BALB/C nude mice (aged 5 weeks) were obtained from Zhuhai BesTest Bio-Tech Co., Zhuhai, China, and 4T1 (Mus musculus breast cancer) cells were cultured in an RPMI-1640 medium supplemented with 10% fetal bovine serum, and 1% penicillin-streptomycin. The tumor cells were injected by approximately concentrating 2 × 10^6^ cells in 0.1 mL of phosphate-buffered saline. Orthotopic breast cancer models were established by injecting 4T1 cells in the mammary fat pads of nude mice. The tumor volume (V) was measured by ultrasound B-mode imaging with digital calipers calculated as V = π/6× a × b × c (a, b, and c refer to width, axial length, and vertical length of tumor). When the tumors’ volumes reached the value of 200 mm^3^, experimental data were collected. (Figure 3)

### 2.4. Tumors’ Subharmonic Scattering Acquisition

The customized ultrasound system was used to acquire data in the in vivo experiment. The mice were anesthetized with inhaled isoflurane (concentration 1.5%, flow 800 mL/min) while lying on a heating pad. Degassed coupling gel was applied on the tumor to minimize the acoustic impedance mismatch between the transducer and tissue to maximize the acoustic transmission. Then, the transducer with a focal depth of 2.0 cm was fixed at the largest transverse cross-section of the tumor. A bolus of 40 µL diluted Sonazoid suspension (dilution 1:20) was injected through the tail vein three times and was followed by a 0.1 mL saline flush. The concentration was 1.6 × 10^−2^ μL/mL. The optimal acoustic pressure was selected on the basis of the in vitro experiment results. The contrast harmonic imaging (HI) mode was chosen to acquire the RF data for five seconds. The interval between each injection was at least 10 min to ensure contrast agent elimination.

### 2.5. IFP Measurement 

The tumor IFP was measured using a tissue fluid pressure monitor (Mode 295-1, Stryker, Kalamazoo, MI, USA) [11] after the ultrasound scan. The device consisted of a needle with a side hole of 3 mm connected to a pressure sensor. The needle filled with saline needed to be inserted into the tissue vertically to make the side hole completely covered by tissue. The indicator of the device should be corrected to zero before measurement. Due to the heterogeneity distribution of IFP in tumors and decreasing IFP in the margin of tumors [4,33], IFP measurements were acquired from the central area of the tumor. Each tumor was measured 3 times in different positions of the central area.

### 2.6. Data Processing

**Figure 4:**
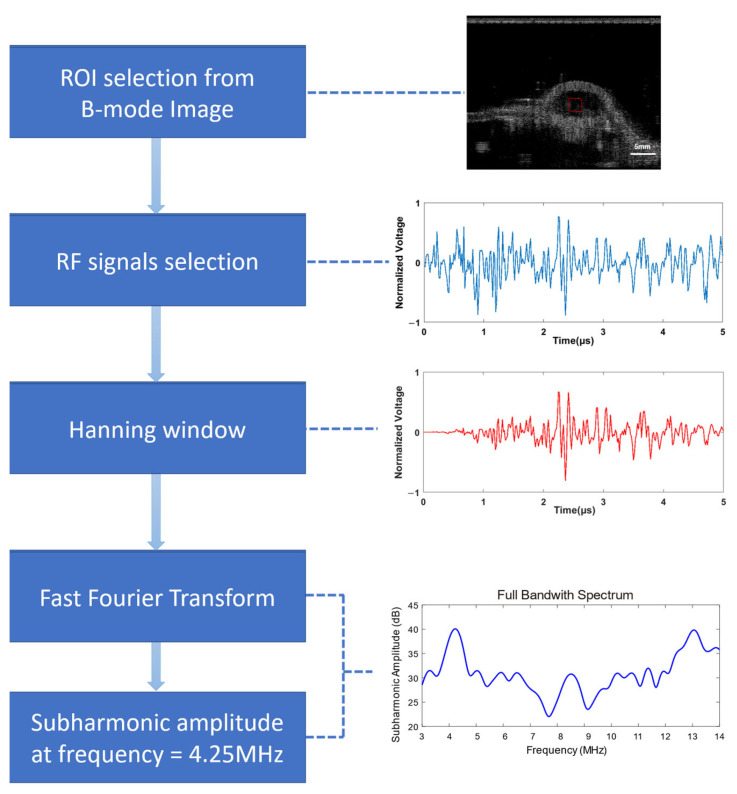
The RF data processing flow (red box, ROI selected).

The RF data were extracted and processed offline on a PC using Matlab (Mathworks Inc., version R2017b, Natick, MA, USA). The region-of-interest (ROI) was selected from the reconstructed B-mode image at the central area of the vascular phantom or the tumor. After adding a Hanning window function on the RF data in the ROI, the amplitude spectrum was further obtained by the fast Fourier transform. The subharmonic amplitude was extracted at the corresponding subharmonic frequency with a bandwidth of 1 MHz on the spectrum (Figure 4). The average amplitude of 20 frames of RF data was recorded. 

### 2.7. Statistical Analysis

The subharmonic amplitude and IFP of various tumor types were presented as the mean ± standard deviation (SD). A linear regression analysis was conducted to compare the relationship between the subharmonic amplitude and IFPs. Cross-validation was performed five times to investigate the stability of the subharmonic estimated pressure among each tumor. All 30 tumors were randomly divided into the model group (n = 20) and validation group (n = 10). Calibration equations were generated from data of the model group to predict the IFPs of the validation group. A paired *t*-test was used to compare the differences between pressures measured by a pressure monitor and the pressures predicted by the subharmonic amplitude using cross-validation. All data and figures were analyzed and presented using SPSS (IBM SPSS, version 25.0, IBM Corporation, Armonk, NY, USA) and GraphPad Prism (GraphPad Software, version 8, Boston, MA, USA). A *p* value < 0.05 was considered statistically significant.

## 3. Results

### 3.1. Resonance Frequency of Sonazoid

**Figure 5:**
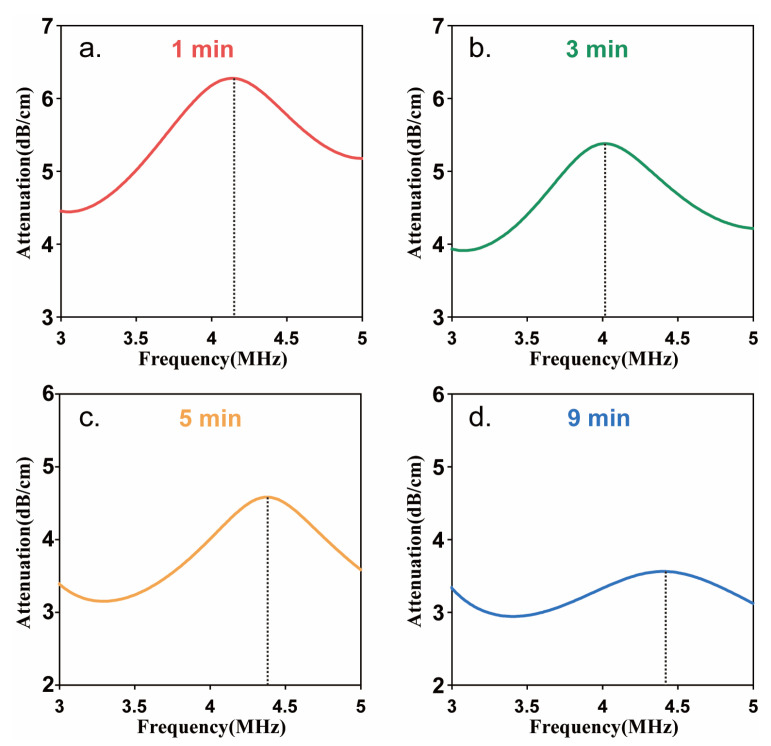
Acoustic attenuation spectra of Sonazoid microbubbles’ suspensions at different injection times. (**a**) 1 min, (**b**) 3 min, (**c**) 5 min, (**d**) 9 min after injection.

In order to obtain the resonance frequency of Sonazoid to make sure the optimal driving frequency generated subharmonic scattering, the acoustic attenuation measurement was first carried out. Figure 5 showed the acoustic attenuation spectra at different times after injecting the Sonazoid suspension. The frequency corresponding to the maximum attenuation coefficient is the resonance frequency. The acoustic attenuation spectra of Sonazoid showed the maximum value of the attenuation coefficient occurred around 4.2 MHz at the time of 1 min after injection. The resonance frequency decreased first and then increased with the injection time and remained in the range of 4–4.5 MHz. Furthermore, the maximum value of the attenuation coefficient decreased continuously with time. Because all the data acquisition was completed within 1 min after the injection of the microbubbles, the optimal driving frequency was determined as 8.5 MHz, approximately equal to twice the resonance frequency at the moment.

### 3.2. Relationship between Subharmonic Amplitude and Ambient Pressure

**Figure 6:**
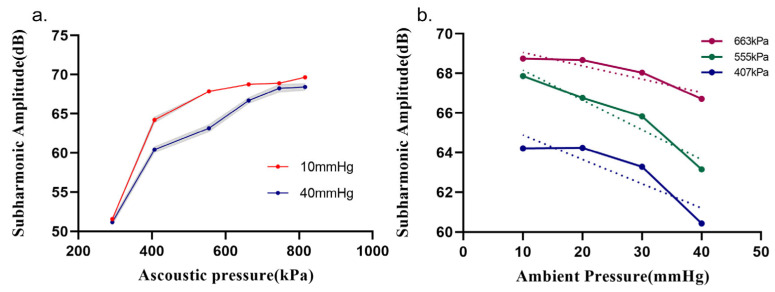
The results of in vitro experiments. (**a**) The amplitude of subharmonic increases with the increased acoustic pressure at ambient pressures of 10 mmHg and 40 mmHg. (**b**) Subharmonic amplitudes vary with ambient pressure at acoustic pressures of 407 kPa (r = −0.883, *p* = 0.117), 555 kPa (r = −0.966, *p* = 0.034), and 663 kPa (r = −0.924, *p* = 0.076).

The acoustic pressure at the focus generated by the linear array was calibrated in the range of 0.29–1.0 MPa (peak negative acoustic pressure), corresponding to mechanical index 0.10–0.34 ($\mathrm{MI}=P_{A}/\sqrt{ƒ})$. According to the results of the acoustic attenuation experiment, the resonance frequency of Sonazoid was in the range of 4–4.5 MHz. The transmitted frequency of 8.5 MHz was applied in subsequent experiments. The acoustic pressures evaluated in the in vitro experiment were 292 kPa, 407 kPa, 555 kPa, 663 kPa, 746 kPa, and 816 kPa. Figure 6a shows that the amplitude of the subharmonic signals increased with the increasing acoustic pressure at ambient pressures of 10 mmHg and 40 mmHg under the above acoustic pressures, respectively. The range of acoustic pressure for exciting subharmonic scattering in this study corresponded to the growth stage (0.3–0.6 MPa) and saturation stage (above 0.6 MPa) consistent with Shi’s findings [16]. As shown in Figure 6a, the subharmonic amplitude of 40 mmHg was always lower than that of 10 mmHg under the same incident acoustic pressure. In addition, the greatest decrease in the subharmonic amplitude reached 4.71 dB at 555 kPa, which indicated an available maximum pressure sensitivity of the subharmonic amplitude. 

In order to investigate the influence of acoustic pressure on the correlation between the subharmonic amplitude and ambient pressure, the subharmonic amplitude vs. ambient pressure curves were further measured for 10 mmHg, 20 mmHg, 30 mmHg, and 40 mmHg under different acoustic pressures, respectively. Figure 6b shows the inverse linear relationships between the subharmonic amplitude and ambient pressure at the acoustic pressures of 407 kPa, 555 kPa, and 663 kPa. As depicted in Table 1, the Pearson’s correlation coefficients of the subharmonic amplitude and ambient pressure were higher than −0.9 when acoustic pressures were 555 kPa, 663 kPa, and 746 kPa, but the result of 663 kPa was not statistically significant (*p* = 0.076). The highest correlation occurred at 555 kPa, with a correlation coefficient of −0.966, and also reached a maximum ambient pressure sensitivity of 0.15 dB/mmHg. When the acoustic pressure was higher than 555 kPa, the difference of subharmonic amplitudes between 10 mmHg and 40 mmHg was decreased with the increase in acoustic pressure. As a result, the sensitivity of the subharmonic amplitude related to the ambient pressure began to decrease from 0.15 dB/mmHg to 0.068 dB/mmHg.

### 3.3. Correlation between Subharmonic Amplitudes and IFPs

**Figure 7:**
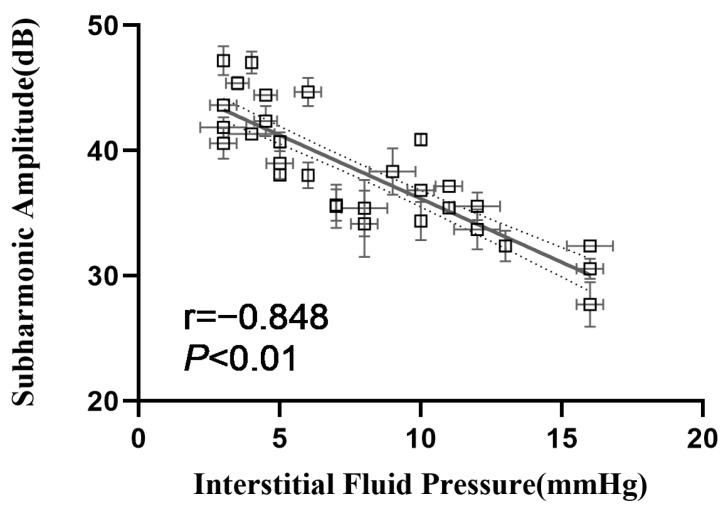
Correlation analysis between tumor IFP and subharmonic amplitudes. The dotted curves on either side of the fitted lines are limits of 95% confidence intervals.

The 30 4T1 tumor IFP models were established, and the range of IFPs for all tumors was 3 to 16 mmHg. This range was consistent with the results of a previous large-sample research study showing that the IFPs of different subcutaneous transplanted tumors of mice ranged from 4.4 to 15.2 mmHg [4]. Because the in vitro experiment demonstrated the maximum ambient pressure sensitivity occurred at an acoustic pressure of 555 kPa, the in vivo experiment was also carried out at 555 kPa. The subharmonic amplitude of tumors decreased with the increase in IFPs. The correlation coefficients between subharmonic amplitudes and IFPs were −0.848 (*p* < 0.01) (Figure 7). To verify the stability of subharmonics’ IFP estimation, cross-validation was performed to obtain the estimated IFPs. As shown in Table 2, the ranges of mean absolute errors and the RMSE between the measured IFP by the standard pressure monitor and the estimated IFP from the subharmonic amplitude in each random group were 1.83 to 2.95 mmHg and 2.25 to 3.60 mmHg. Paired *t*-tests demonstrated no statistical differences between the pressures measured by the pressure monitor and estimated values (*p* > 0.05), which indicated the stability applicability of IFP estimation using subharmonic scattering from UCA microbubbles. 

## 4. Discussion

In our investigation, the ambient pressure sensitivity of Sonazoid’s subharmonic amplitude exceeded that reported in the literature, primarily due to the selection of driving frequency. The choice of the driving frequency is usually based on the specific tissue for the in vivo application. In clinical practice, a low-frequency ultrasound of 1–5 MHz is commonly used for the liver and heart, as they typically require a greater depth of penetration. In order to estimate the portal vein pressure and intracardiac pressure, Forsberg et al. utilized 2.5 MHz ultrasound waves to excite Sonazoid microbubbles and generate subharmonic scattering signals with a frequency of 1.25 MHz [24,25]. In their prior study on Sonazoid, the greatest pressure sensitivity of 0.08 dB/mmHg was attained for the subharmonic amplitude at a drive frequency of 2.5 MHz and an acoustic pressure of 0.35 MPa [17]. In contrast, superficial tumors such as the breast and thyroid require higher ultrasound frequencies for superior imaging resolution. Therefore, we employed acoustic waves with a frequency larger than 7.5 MHz for estimating tumor interstitial fluid pressure. While Sonazoid had a resonance frequency of 4.4 MHz [31], its optimal driving frequency for subharmonic excitation was double the resonance frequency in theory, resulting in an ideal driving frequency of 8.8 MHz to generate subharmonic scattering for assessing the IFP of superficial tumors. Consequently, our current study achieved a high pressure sensitivity of 0.15 dB/mmHg at a driving frequency of 8.5 MHz and an acoustic pressure of 0.55 MPa. In addition, the pressure sensitivities (1.015 dB/mmHg) in the tumor models were significantly higher than those in the vascular phantom. This difference between the in vitro and in vivo experiments was also observed in previous studies for Definity, Sonazoid, and SonoVue [25,31,34]. The main reason may be that the microbubbles exhibited more violent oscillation, more destruction, and produced more nonlinear scattering at body temperature (25 °C) [35].

Tumor heterogeneity makes it possible for the same tumor type to exhibit different biological behaviors. The 4T1 orthotopic breast cancer models in this study were manifested as an IFP range of 3–16 mmHg, which will influence the perfusion of tumors. Whether the subharmonic-based IFP estimation is dependent on individual differences is important for the promotion of the technique. In our study, the optimal acoustic pressure of 555 kPa in the in vitro experiment was applied to the tumor IFP model. Excellent correlations and high pressure sensitivities between the tumor IFP and subharmonic amplitudes extracted from tumor tissues were demonstrated (r = −0.848, *p* < 0.05, pressure sensitivity: −1.015 dB/mmHg). Moreover, a five-time cross-validation study showed relatively small errors (mean absolute error less than 2.95 mmHg, RMSE less than 3.60 mmHg). These results suggest the stability and universality of subharmonic-amplitude-based IFP estimation in tumor models.

There are some limitations to this study. The optimal acoustic pressure, at which the subharmonic amplitudes were sensitive to ambient pressures, needed to be processed off-line in every experimental setting. The clinical application of this technique required commercial transducers and ultrasonic machines equipped with a real-time data processor. An acoustic pressure optimization program should be further developed to compensate the attenuation from acoustic wave propagation in the tissue. The skin thickness was about 1 mm. As a result, the attenuation of the transmitted 8.5 MHz acoustic wave in the skin can be negligible before reaching the subcutaneous transplanted tumor, and depths of superficial tumors in humans are various. The attenuation caused by acoustic wave propagation in the tissue is affected by tissue type and depth. The attenuation coefficient of fat and breast is 0.48 dB/cm/MHz and 0.75 dB/cm/MHz [36]. The depth of the breast lesions ranges from less than 1 cm to more than 5 cm. Patients with fatty breast composition, usually with lesion depth greater than 5 cm, will have more than 20 dB attenuation at 8.5 MHz. So, the output acoustic pressure should be compensated according to the depth of the lesion to ensure that the acoustic pressure applied to the lesion is optimal. Another limitation was that the range of IFP models established in mice was limited (3–16 mmHg), which was lower than the reported IFP of human tumors. Despite the fact that the effectiveness of subharmonic amplitudes in estimating ambient pressure in a range of 10–40 mmHg has been demonstrated in vitro, a study with a wider IFP range and larger animal sample needs to be performed to accumulate evidence and data for improving this technology. In addition, subsequent clinical studies will enroll breast cancer patients to evaluate the relationship between subharmonics and tumor grade, treatment outcome, and prognosis.

## 5. Conclusions

In this study, we obtained the optimal acoustic pressure and driving frequency in vitro for UCA Sonazoid microbubbles’ subharmonic scattering with ambient pressure and verified the excellent interstitial fluid pressure correlation and sensitivity using these parameters in tumor models. The results of the further cross-validation demonstrated the robustness of subharmonic-amplitude-based tumors’ IFP estimation. Further in-depth studies will be conducted to improve the ultrasonic system for monitoring the efficacy of chemotherapy. Overall, this study suggested that subharmonic-amplitude-based IFP estimation provided a promising biomarker for the noninvasive and accurate evaluation of tumor microenvironments.

## Figures and Tables

**Table 1:** The ambient pressure sensitivity of the subharmonic amplitude and correlation analysis of the in vitro experiments.

Acoustic Pressure (kPa)	MI ^1^	Sensitivity (dB/mmHg)	Pearson’s r	*p* Value
292	0.100	0.012	−0.862	0.138
407	0.140	0.123	−0.883	0.117
555	0.190	0.150	−0.966	0.034
663	0.227	0.068	−0.924	0.076
746	0.256	0.029	−0.999	0.001
816	0.280	0.028	−0.569	0.431

^1^ MI = mechanical index.

**Table 2:** Comparing errors between the pressures measured by pressure monitor and the pressures predicted by the subharmonic amplitude using cross-validation.

Random Grouping	Mean Absolute Error (mmHg)	SD (mmHg)	Range (mmHg)	RMSE ^1^ (mmHg)	*p* Value
1	2.09	1.56	[0.06, 4.93]	2.52	0.193
2	2.26	1.24	[0.19, 4.06]	2.54	0.098
3	1.83	1.46	[0.30, 4.16]	2.25	0.192
4	2.95	2.28	[0.16, 6.25]	3.60	0.458
5	2.11	1.46	[0.04, 4.02]	2.59	0.400

^1^ RMSE = rooted mean squared error.

## Data Availability

The data that support the findings of this study are available from the corresponding author upon reasonable request.
